# Why pair? Evidence of aggregative mating in a socially monogamous marine fish (*Siganus doliatus*, Siganidae)

**DOI:** 10.1098/rsos.150252

**Published:** 2015-09-16

**Authors:** Rebecca J. Fox, David R. Bellwood, Michael D. Jennions

**Affiliations:** 1 School of Life Sciences, University of Technology Sydney, Ultimo, New South Wales 2007, Australia; 2 College of Marine and Environmental Sciences and ARC Centre of Excellence for Coral Reef Studies, James Cook University, Townsville, Queensland 4811, Australia; 3 Division of Evolution Ecology and Genetics, Research School of Biology, Australian National University, Canberra, Australian Capital Territory 2601, Australia

**Keywords:** coral reef fish, pairing, reproduction, social systems, spawning aggregation

## Abstract

Many species live in stable pairs, usually to breed and raise offspring together, but this cannot be assumed. Establishing whether pairing is based on mating, or an alternative cooperative advantage, can be difficult, especially where species show no obvious sexual dimorphism and where the act of reproduction itself is difficult to observe. In the tropical marine fishes known as rabbitfish (Siganidae), half of extant species live in socially monogamous, territorial pairs. It has been assumed that partnerships are for mating, but the reproductive mode of pairing rabbitfish is currently unconfirmed. Using passive acoustic telemetry to track movements of fishes belonging to one such species (*Siganus doliatus*), we provide the first evidence that paired adult fish undertake highly synchronized migrations with multiple conspecifics on a monthly cycle. All tagged individuals migrated along the same route in three consecutive months and were absent from home territories for 2–3 days just after the new moon. The timing and directionality of migrations suggest that *S. doliatus* may form spawning aggregations, offering the potential for exposure to multiple reproductive partners. The finding raises fundamental questions about the basis of pairing, mate choice and partnership longevity in this family.

## 1. Introduction

The observation of a stable pair bond in nature tends to lead to an initial presumption that the partnership is for mating [1,2]. However, pairs can also form as cooperative alliances to acquire access to resources or commodities [3–5]. Explaining why any partnership in nature forms and persists is crucial to understanding the evolution of cooperation and sociality, yet determining the basis of the apparently simple phenomenon of pairing can raise challenges. Determining the basis of pairing in marine fishes presents particular challenges, both theoretical and practical. On the theoretical side, there is the struggle to explain monogamy in the context of life cycles containing a dispersive larval phase (i.e. no post-mating selection for biparental care). Alternative hypotheses to explain the basis of pairing typically invoke either reproductive advantages (e.g. low population density so that stable pairing increases efficiency by reducing the time to locate a mate) or non-mating benefits such as greater access to resources by pairs than individuals (stronger territory defence, increased feeding efficiency or increased predator vigilance) [6,7]. On the practical side, sexing of individuals is hindered in the many species of marine fishes that do not exhibit obvious external sexual dimorphism, while the marine environment poses challenges for observations of mating behaviour, particularly in larger, mobile species. Therefore, data confirming whether pairings consist of a male and a female that mate together are often lacking and the basis of pairing in many species of marine fishes remains unresolved (but see [8]).

The rabbitfishes (Siganidae) are a family of tropical and subtropical marine fishes distributed across the Indo-Pacific, Red Sea and Mediterranean. There is a within-family dichotomy between species that form loosely associative schools as adults and those that form stable, territorial pairs [9]. For the 14 species that pair as adults, the evolutionary advantage of pairing has yet to be established. It has been assumed that pairs consist of a male and female that mate together over multiple reproductive seasons, but with species exhibiting no obvious external sexual dimorphism and with next to nothing known about their reproductive behaviour (‘Reproduction in permanently pairing species not recorded’ [10], p. 3627), these assumptions predominantly remain untested. The presupposition that pairs always consist of a male and female has already been invalidated by the discovery of same-sex pairings within populations of rabbitfish [11]. However, it is still not known whether the heterosexual pairings that make up the majority of the population are breeding together on territories and therefore whether pairing is still based on the benefits of sequestering a mating partner, as in the Laysan albatross where pairs are for mating but same-sex partnerships exist [12,13]. The aim of the current study was therefore to investigate the nature of the mating system of the pairing species of rabbitfish, in particular the pencil-streaked rabbitfish, *Siganus doliatus*, that inhabits coral reefs of the western Pacific and northeast Australia [9]. When individuals reach a size of approximately 7 cm, they form strong pair bonds. Pairs have a consistent home range [11] and appear to defend a territory, with contact between pairs typically avoided and acts of aggression (chasing) and displays observed (R.J.F., personal observation). Pairs are size-assorted and there is no obvious external sexual dimorphism. Nothing is currently known about the species' reproductive behaviour in the wild [9,10]. We used passive acoustic telemetry to monitor the movements of *S. doliatus* over a six-month period spanning the Austral spring and summer and including the months of peak fish recruitment on the Great Barrier Reef [14]. Based on recruitment patterns, and taking into account the constraints of finite acoustic transmitter battery life (167 days maximum), it was hoped that the study period would maximize the likelihood of detecting behaviour relating to reproductive events. Our aim was to examine whether individuals showed evidence of changes in movement pattern that could be indirectly related to reproduction, or whether they would remain within their home territory over the entire period, suggesting that mating was taking place within pairs on territories.

## 2. Material and methods

The study was carried out in waters surrounding Orpheus Island (18°35′ S, 146°20′ E), one of the inner-shelf islands of the Great Barrier Reef, Australia. A linear array of 13 acoustic receivers (Vemco VR2W, 69 kHz frequency, 308 × 70 mm; Amirix Pty Ltd, Nova Scotia, Canada) was deployed along a 3 km stretch of continuous coral reef habitat on the western side of the island (electronic supplementary material, figure S1). Eight adult fish belonging to the pair-forming species of rabbitfish *S. doliatus* were caught from locations within the array and tagged with a coded acoustic transmitter (Vemco V9–1L, 24×9 mm; Amirix Pty Ltd) inserted into the peritoneal cavity while under anaesthesia (0.1 g l^−1^ salt water solution of MS-222). All fish were observed within a pair prior to capture, but only one individual from each pair was tagged to ensure independence between observations. Individuals were released back at their point of capture so that they would have the opportunity to re-form original pairings (table 1). Fish released concurrently did not form partnerships with each other, as evidenced by the lack of identical detection patterns within the dataset.

**Table 1.** Summary of *S. doliatus* individuals (SD1–SD8) acoustically tagged and tracked in the study. TL, total length; SL, standard length.

fish ID	location caught and released	social status	size (TL) (cm)	size (SL) (cm)	mass (g)	duration tracked (days)
SD1	18°36′22.19^′′^ S, 146°29′19.51^′′^ E	pair, partner not captured released with SD2 and SD4	21.3	16.7	164.8	167
SD2	18°36′22.19^′′^ S, 146°29′19.51^′′^ E	pair, partner not captured released with SD1 and SD4	22.2	17.3	182.1	167
SD3	18°35′57.60^′′^ S, 146°29′23.02^′′^ E	pair, partner not captured released alone	23.9	18.8	225.6	167
SD4	18°36′22.19^′′^ S, 146°29′19.51^′′^ E	pair, partner not captured released with SD1 and SD2	24.5	19.3	255.7	167
SD5	18°36′26.40^′′^ S, 146°29′20.01^′′^ E	pair, partner not captured released with SD8	22.0	16.8	178.4	167
SD6	18°36′26.40^′′^ S, 146°29′20.01^′′^ E	pair, partner not captured released with SD7	23.5	17.8	190.5	167
SD7	18°36′26.40^′′^ S, 146°29′20.01^′′^ E	pair, partner not captured released with SD6	24.8	18.9	239.6	167
SD8	18°36′26.40^′′^ S, 146°29′20.01^′′^ E	pair, partner not captured released with SD5	23.9	18.1	206.4	167

Fish were tracked for 167 days (duration of transmitter battery life) over the Austral spring and summer (August–February). The VR2W acoustic receivers logged the presence (time and date stamp) of tagged individuals passing within range. Data were downloaded at eight-week intervals and logged detection sequences were viewed in VUE software v. 1.4. An individual's home territory location was defined as the receiver on which most detections were recorded. Detection ranges were calculated for all receivers individually and were non-overlapping, meaning that we were able to distinguish between movements made by an individual within its home territory location and movements outside the home territory to a neighbouring receiver.

## 3. Results

**Figure 1.**
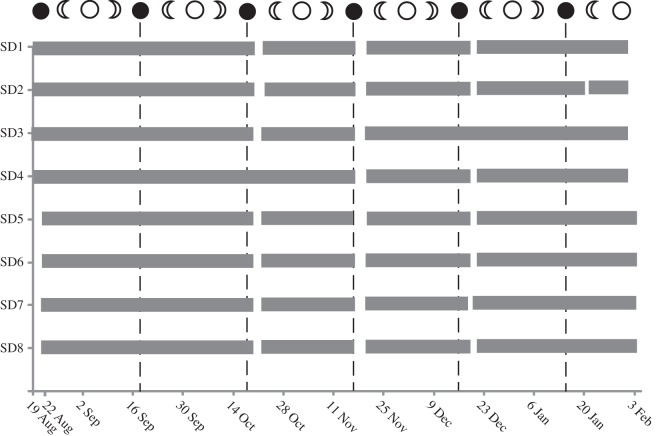
Presence of tagged individuals of *S. doliatus* recorded within acoustic monitoring array, Pioneer Bay, Orpheus Island. Grey bars indicate periods where individuals were detected within the array. Presence is shown relative to lunar phases: 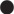, new moon; 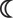, first quarter; 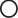, full moon; 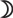, last quarter. Moon phase dates and times from http://museumvictoria.com.au/planetarium/discoverycentre/moon-phases/.

**Figure 2.**
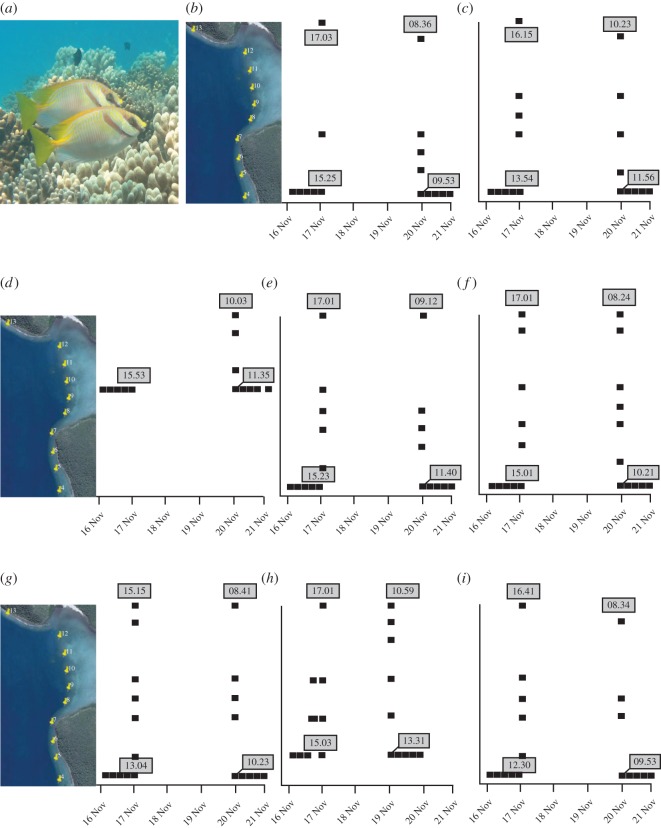
Abacus plots of receiver detections for individuals of the species (*a*) *S. doliatus*. (*b*–*i*) SD1–SD8 from 16 to 21 Nov. Detections (black squares) are displayed alongside schematic of receiver array and show northward movement of fishes out of Pioneer Bay and subsequent return to home territories. Timings of (i) departure from home territory, (ii) departure from Pioneer Bay (last detection on northern-most receiver), (iii) arrival back to Pioneer Bay (next subsequent detection), and (iv) arrival at home territory, are given. Similar plots for Oct and Dec are provided in the electronic supplementary material.

All eight tagged fish were detected daily within the 3 km of monitored coastline, with the key exception of three periods: 20–22 October, 17–20 November and 19–21 December. For each of these months, the dates on which fish were not detected within the array fall 1–4 days after the new moon (figure 1). For every individual, the period of absence was preceded by an identical pattern of detections. Fish left their home territory and moved northward, with detections recorded on receivers in sequence until they reached the northern-most receiver PB13 (figure 2). Many of the final detections on PB13 for a particular month were within minutes of each other (electronic supplementary material, figure S2). After 2–3 days during which no individuals were detected in Pioneer Bay, fish were again registered at PB13 swimming southward, with detections recorded at receivers in reverse sequence until each individual reached its home territory (figure 2). All eight individuals migrated in October, November and December, except SD4 who did not migrate in October, and SD3 who did not migrate in December (figure 1). One individual (SD2) migrated in January, but this was the shortest recorded absence (20.5 h).

The median time spent away from the home territory varied from 48.2 h in October to 68.3 h in November and 50.3 h in December (electronic supplementary material, figure S3). The total time spent away per migration was consistent across tagged individuals, although SD2 returned a day later than all others in October, and SD7 returned a day earlier than all other tagged individuals in both November and December (electronic supplementary material, figure S3).

## 4. Discussion

Long-term monitoring of the movement patterns of *S. doliatus* revealed that in October, November and December (and January for one individual), tagged individuals migrated in a northerly direction and beyond the northern range of the acoustic array. The movements were closely synchronized, occurred only in certain months, and were tied to a lunar cycle (in this case, shortly after the new moon). Based on documented patterns of reproductive aggregations in coral reef fishes [15], we conclude that these movements are strongly suggestive of migration to spawning aggregation sites and therefore that *S. doliatus*, like its schooling congenators [16–18], migrates to breed at aggregations some distance from their home territory. Our results provide the first empirical evidence of reproductive behaviour in a pairing species of rabbitfish and they provide strong support for previous anecdotal reports of spawning aggregations in pairing species [15,16]. In this study, fish were last detected at a point up to 2 km from their home territories. With their final spawning destination(s) as yet unknown, this represents a minimum migration distance for *S. doliatus* and compares with reported migrations of 3.3 km to spawning aggregation sites for the schooling species *S. sutor* in southern Kenya [19]. Many of the final detections on PB13 for a particular month were within minutes of each other, suggesting that some fish were swimming in aggregation by this point along the migration route. The observation mirrors the ‘pre-spawning’ aggregating behaviour recorded in related schooling species of rabbitfish [18–20] (where fish congregate together at a location before migrating *en masse* to their final destination), and it provides further evidentiary support for the assertion that the observed migrations were for reproduction. The total time spent away from home territories by *S. doliatus* in this study was between 48 and 68 h depending on month. Subtracting from that period, the total time spent in transit, which we know to be a minimum of 6 h for individuals recorded moving north and then south within the array, suggests that maximum aggregation residency times will be of the order of 40–62 h, and are likely to be less. These durations are similar to the spawning residency times recorded for the schooling species *S. sutor* of up to 2 days at an aggregation site in southern Kenya [19] and 0.7–3.7 days at sites in the Seychelles, with residency time decreasing in the second half of the spawning season [18].

As well as providing the first empirical evidence of synchronized migrations in relation to lunar cycles in pairing rabbitfish that is consistent with observed aggregative spawning behaviour among schooling rabbitfish species, our results also raise interesting new questions about the reproductive behaviour of the pairing species of rabbitfish, and the evolutionary forces selecting for pairing within this family of marine fishes. The suggestion that pairing species may also migrate to spawn at aggregations poses a challenge to assumptions relating to (i) the longevity of pair bonds in rabbitfish and (ii) pairing in this family being favoured by the benefits of monogamous mating. Reproductive aggregations, by their nature, offer exposure to multiple partners, raising the possibility that social partners may not necessarily be mating partners and that mates could be traded at each reproductive event. Until now, it has been assumed that observed rabbitfish pairings were long-term partnerships, but aggregative spawning means that individuals could potentially return from each aggregation with a different social partner, resulting in pairings being more labile than currently assumed. From the current study, we were unable to determine whether pairs migrate to and return from the aggregation together (only one member of each pair was tracked to avoid pseudo-replication), but our results did show that all tagged individuals returned to an area of reef within the detection range of the same receiver each month. The fact that fish were returning to the same area of reef after each migration makes it more likely that there is an element of stability in these partnerships. Tests of the longevity of pairings, including confirming whether an individual returns to its home territory with the same partner after each spawning event, should now be a priority.

The suggestion that pairing rabbitfish, like their schooling relatives, may migrate to reproduce at aggregations also throws into question the assumption that pair formation in rabbitfish is driven by the benefits of mating monogamously. If pairs were for reproduction, why would females incur the risks associated with a lengthy migration when the opportunity exists to breed on their territory with their social partner? It is possible that increased early offspring survival due to key benthic (substrate type) or oceanographic features might drive females to aggregate at particular sites, with males having no choice but to follow and that mating still takes place within social pairs at the aggregation site. Rabbitfish, rather than releasing gametes into the water column for pelagic fertilization, ‘scatter’ negatively buoyant, sticky eggs onto the benthos [21]. The larvae hatch after 25–32 h and are dispersed into the pelagic zone by prevailing currents [21]. Sites may therefore be selected so as to provide suitable benthic laying sites, or to maximize offshore current dispersal. Alternatively, the survival advantages from spawning in a pair with many other pairs around may drive sexually monogamous pairs to aggregate. Equally, though, females may migrate because aggregations increase their ability to mate with, and/or to use sperm competition to bias paternity towards males other than their social partner. If spawning aggregations of pairing rabbitfish are on a scale equivalent to those of their schooling congenators, the impossibility of observing specific individuals within an aggregation of several thousand fishes (J. Bijoux, personal communication) makes it unlikely whether we can test an individual spawns only with their social partner at such reproductive events. Testing alternative hypotheses (e.g. enhanced feeding efficiency, increased predator vigilance) for the evolutionary advantage of pairing in this family is likely to represent the most productive way forward in determining whether pairing is for reproduction in rabbitfish. However, our findings do add to a growing body of evidence for a non-reproductive basis for pairing in the Siganidae. Specifically, the recent discoveries that same-sex pairings, both male–male and female–female, are common within our study population (25% of pairs sampled were same-sex [11]), and that pairs of at least three rabbitfish species appear to coordinate vigilance when foraging [22] suggest that there may be a non-reproductive basis to pairing within the Siganidae. Empirical tests of these alternative hypotheses are now required to identify the factors that promoted a transition from schooling to pair formation on territories [23] within this major family of marine fishes.

## Supplementary Material

Figure S1: Map of study site Figure S2: Abacus plots of receiver detections giving detail of monthly northward migration out of Pioneer Bay, Orpheus Island and corresponding southward migration back to home territory for all tagged Siganus doliatus (SD1-SD8). Figure S3: Duration of monthly migrations undertaken by eight individual Siganus doliatus outside of their home territories within Pioneer Bay, Orpheus Island, Australia.

## Data accessibility

Our data files have been uploaded to Dryad: http://dx.doi.org/10.5061/dryad.29gb5.
