# Supplementary material for: Why pair? Evidence of aggregative mating in a socially monogamous marine fish (Siganus doliatus, Siganidae)
Source: R Soc Open Sci. 2015 Sep 16;2(9):150252. doi: 10.1098/rsos.150252 (PMC4593683; doi:10.1098/rsos.150252)
Supplement: Figure S1: Map of study site Figure S2: Abacus plots of receiver detections giving detail of monthly northward migration out of Pioneer Bay, Orpheus Island and corresponding southward migration back to home territory for all tagged Siganus doliatus (SD1-SD8). Figure S3: Duration of monthly migration [file rsos150252supp1.pdf]

Supplementary Material: Why pair? Evidence for aggregative mating in a socially monogamous marine fish (*Siganus doliatus*, Siganidae)

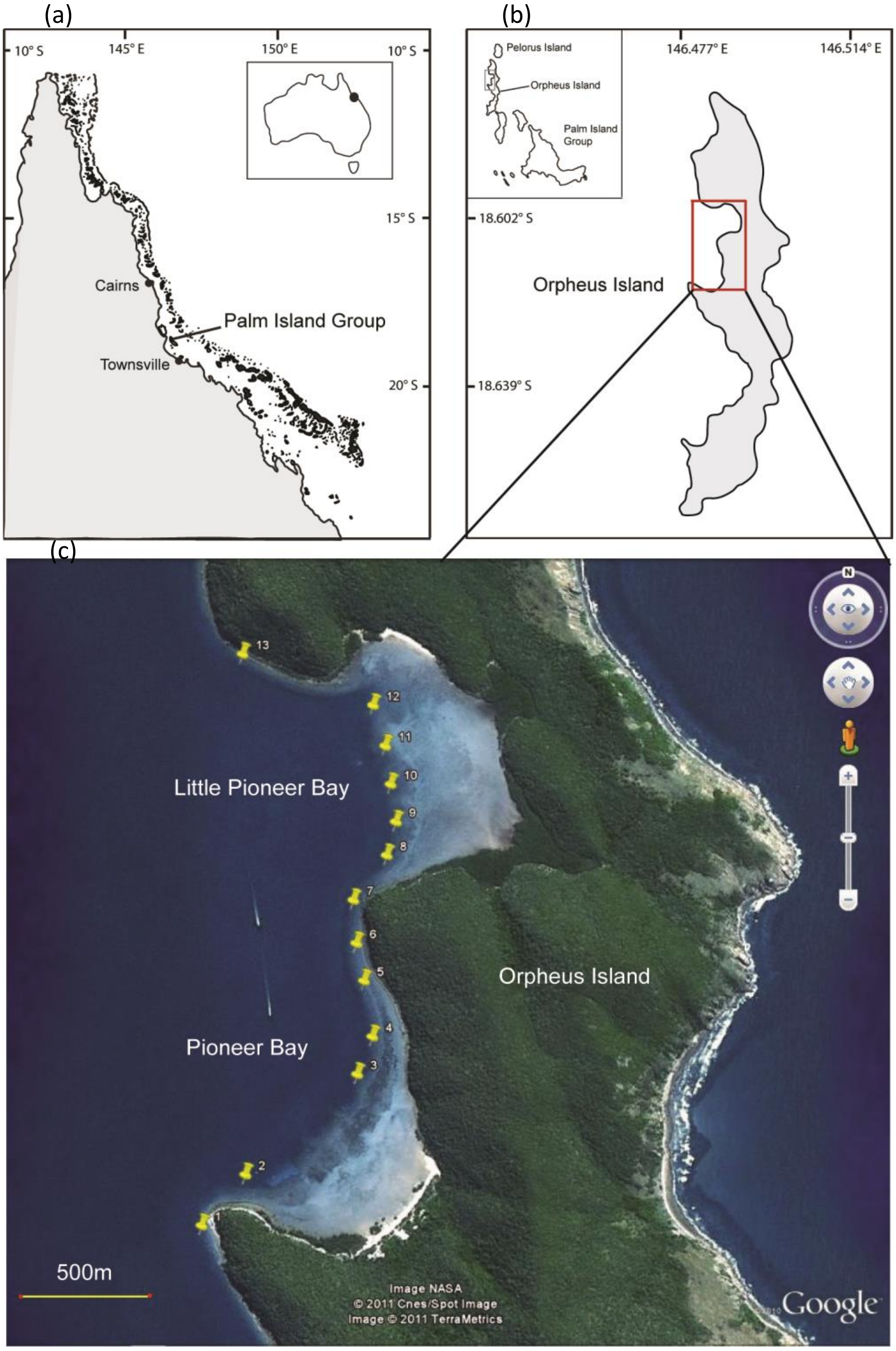

Figure S1: Map of study site showing (a) the location of Palm Island Group within the Great Barrier Reef, 11km east of the Australian mainland, (b) position of Orpheus Island relative to neighbouring islands within the Palm Island group (inset) and location of study site (red rectangle) on western side of Orpheus Island. (c) Map of study site, Pioneer Bay, Orpheus Island. Yellow pins indicate the position of remote acoustic receivers in linear array.

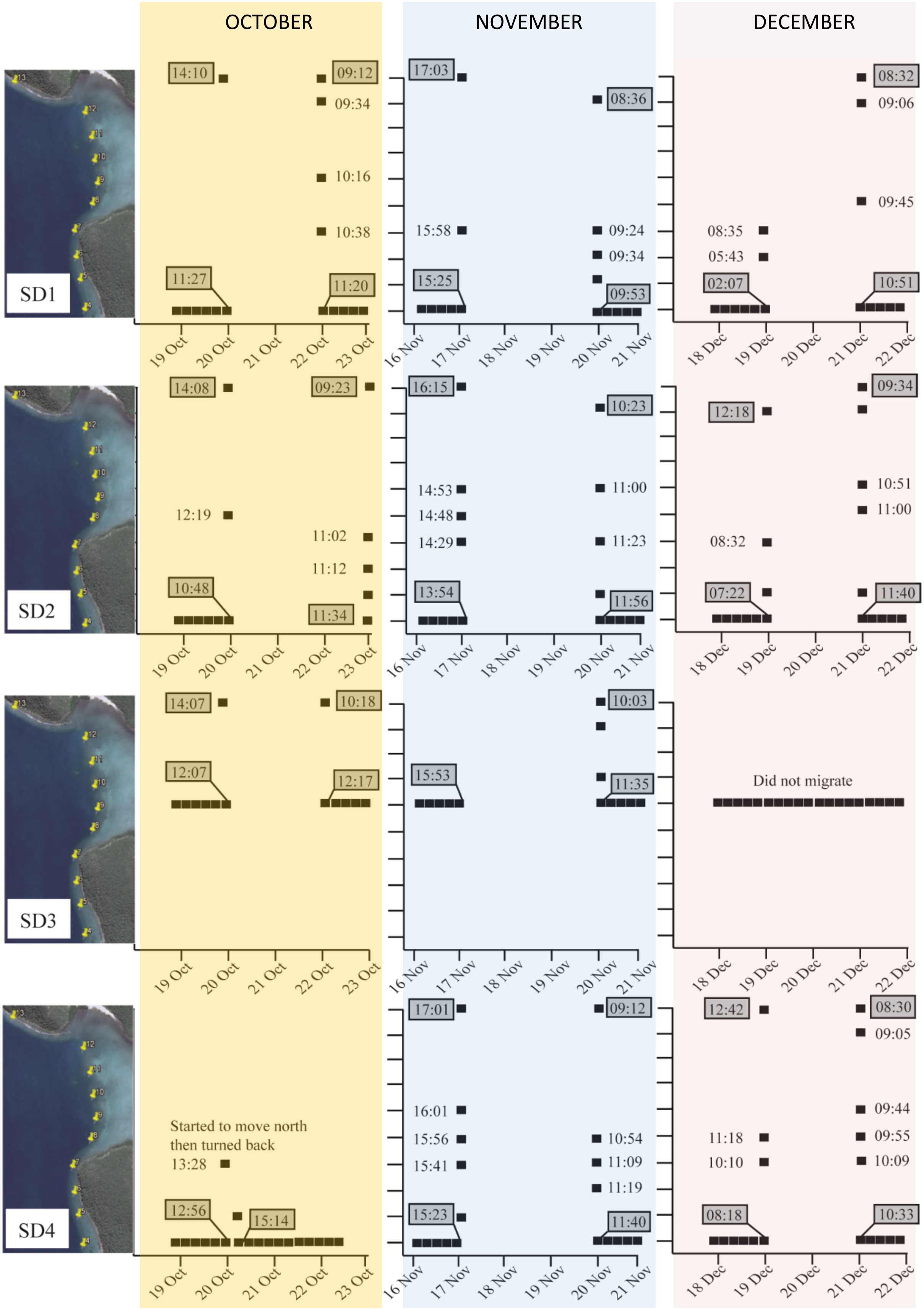
OCTOBER
NOVEMBER
DECEMBER
SD1
14:10
09:12
09:34
10:16
10:38
11:27
11:20
17:03
08:36
15:58
09:24
09:34
15:25
09:53
08:32
09:06
09:45
08:35
05:43
02:07
10:51
19 Oct
20 Oct
21 Oct
22 Oct
23 Oct
16 Nov
17 Nov
18 Nov
19 Nov
20 Nov
21 Nov
18 Dec
19 Dec
20 Dec
21 Dec
22 Dec
SD2
14:08
09:23
12:19
11:02
11:12
10:48
11:34
16:15
10:23
14:53
14:48
14:29
11:00
11:23
13:54
11:56
09:34
12:18
10:51
11:00
08:32
07:22
11:40
SD3
14:07
10:18
12:07
12:17
10:03
15:53
11:35
Did not migrate
SD4
Started to move north
then turned back
13:28
12:56
15:14
17:01
09:12
16:01
15:56
15:41
10:54
11:09
11:19
15:23
11:40
12:42
08:30
09:05
09:44
11:18
09:55
10:10
10:09
08:18
10:33

(...../cont.)

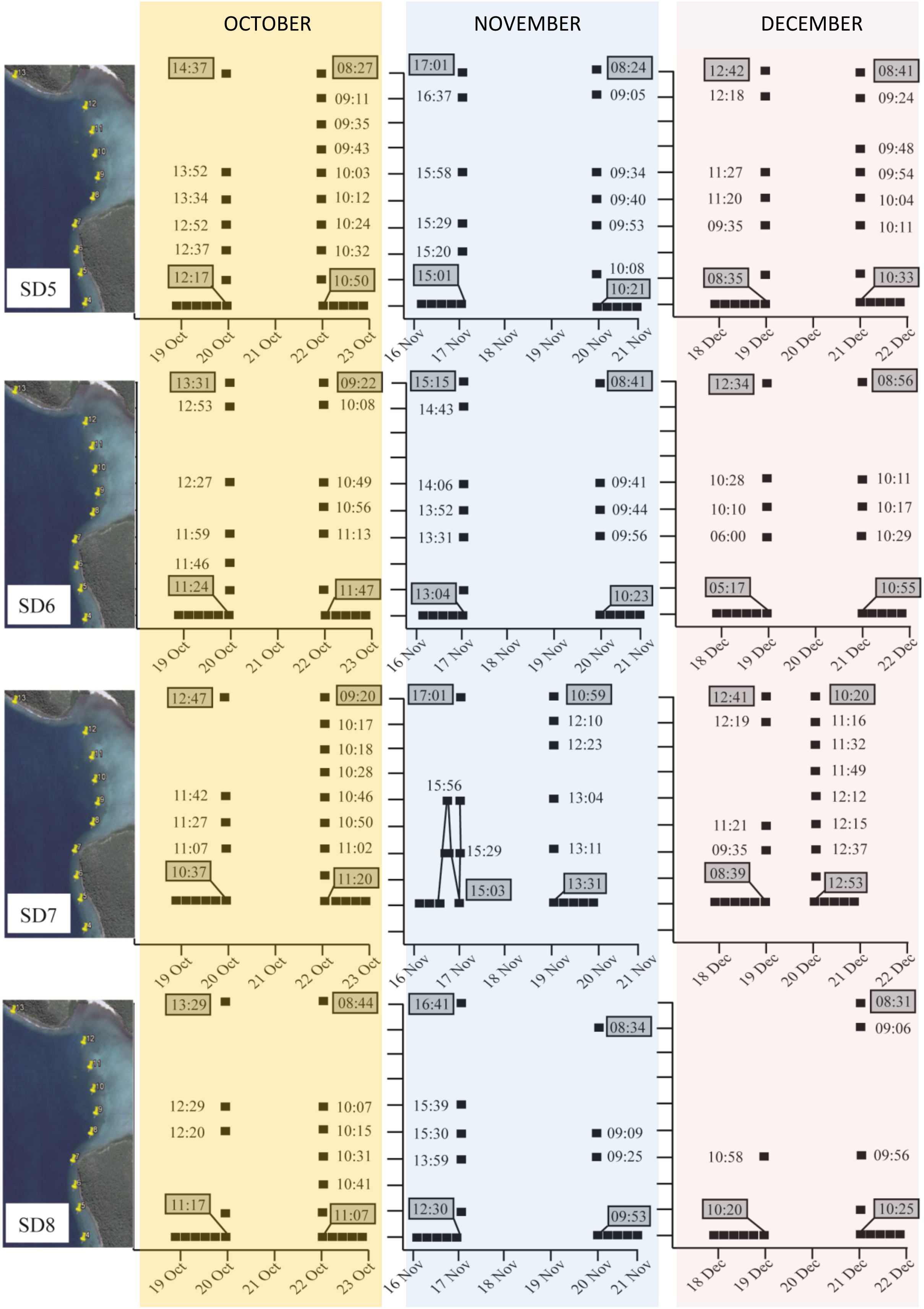

Figure S2: Abacus plots of receiver detections giving detail of monthly northward migration out of Pioneer Bay, Orpheus Island and corresponding southward migration back to home territory for all tagged *Siganus doliatus* (SD1-SD8). The timing of each individual's departure from home territory, departure from Pioneer Bay (defined as last detection on northernmost receiver), arrival back to Pioneer Bay (defined as next subsequent detection on northernmost receiver) and arrival back at home territory are presented for months in which migration was undertaken.

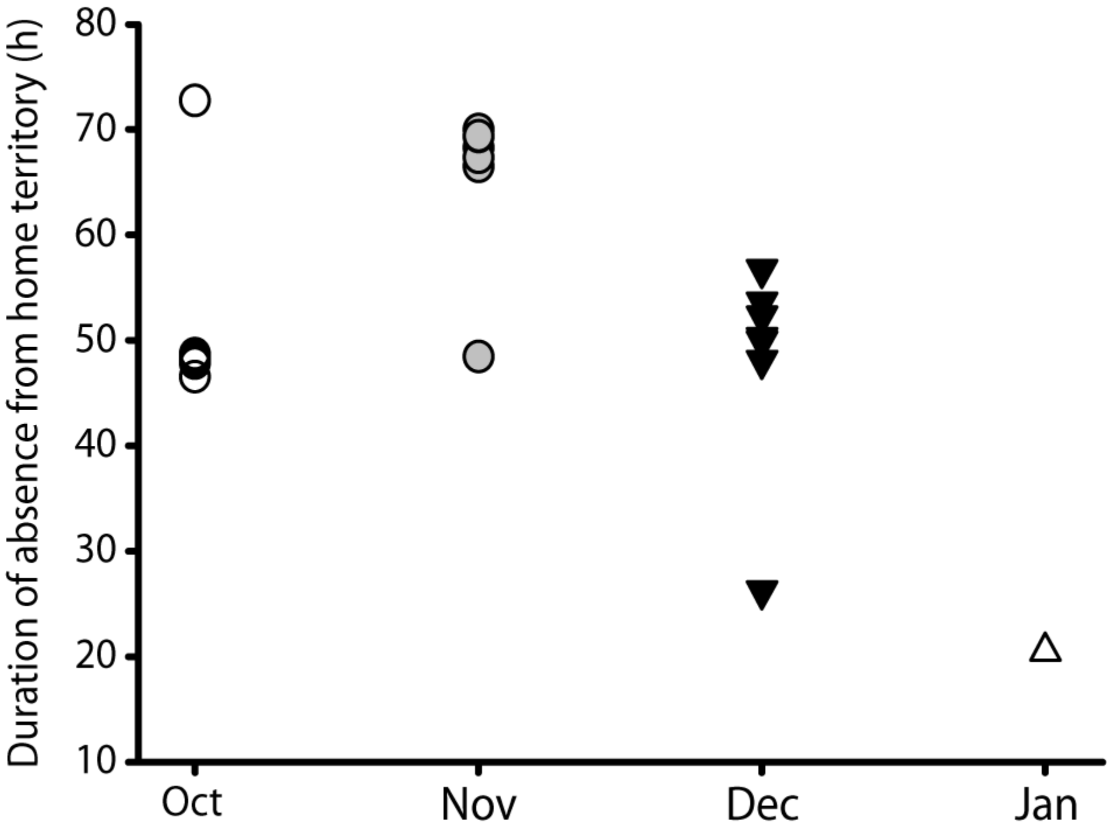

Figure S3: Duration of monthly migrations undertaken by eight individual *Siganus doliatus* outside of their home territories within Pioneer Bay, Orpheus Island, Australia. Migrations were undertaken in the months of October, November, December and January, spanning the Austral summer (just one of the eight tagged individuals undertook a migration in the January). Transmitter battery life expired prior to the date of the February new moon.
